# Potential anatomical triggers for plan adaptation of cervical cancer external beam radiotherapy

**DOI:** 10.1007/s13246-024-01473-2

**Published:** 2024-08-08

**Authors:** Rhianna Brown, Lois Holloway, Annie Lau, Karen Lim, Pereshin Moodaley, Peter Metcalfe, Viet Do, Dean Cutajar, Amy Walker

**Affiliations:** 1 Liverpool and Macarthur Cancer Therapy Centres, Sydney, NSW Australia; 2 Ingham Institute of Applied Medical Research, Sydney, NSW Australia; 3 Centre for Medical Radiation Physics, University of Wollongong, Wollongong, NSW Australia; 4 South West Sydney Clinical Campuses, University of New South Wales, Sydney, NSW Australia; 5 Institute of Medical Physics, University of Sydney, Sydney, NSW Australia; 6 St George Cancer Care Centre, St George Hospital, Kogarah, NSW Australia

**Keywords:** Adaptive radiotherapy, MRI, Cervical cancer, Anatomical changes, Dosimetric changes

## Abstract

This study aimed to identify potential anatomical variation triggers using magnetic resonance imaging for plan adaption of cervical cancer patients to ensure dose requirements were met over an external beam radiotherapy course. Magnetic resonance images (MRIs) acquired before and during treatment were rigidly registered to a pre-treatment computerised tomography (CT) image for 11 retrospective cervix cancer datasets. Target volumes (TVs) and organs at risk (OARs) were delineated on both MRIs and propagated onto the CT. Treatment plans were generated based on the pre-treatment contours and applied to the mid-treatment contours. Anatomical and dosimetric changes between each timepoint were assessed. The anatomical changes included the change in centroid position and volume size. Dosimetric changes included the V30Gy and V40Gy for the OARs, and V95%, V100%, D95% and D98% for the TVs. Correlation with dosimetric and anatomical changes were assessed to determine potential replan triggers. Changes in the bowel volume and position in the superior-inferior direction, and the high-risk CTV anterior posterior position were highly correlated with a change in dose to the bowel and target, respectively. Hence changes in bowel and high-risk CTV could be used as a potential replan triggers.

## Introduction

During the treatment of cervical cancer, anatomical changes can occur to the target volumes (TVs) and organs at risk (OARs) [1–4]. These anatomical changes can be observed as changes in the position, shape and size of the volumes, often attributed to organ motion in the form of bladder and rectal filling, and/or as a result from the treatment itself [1–4]. Delivered dose to the TVs and OARs can be impacted by these changes [5] which can be quantified through changes to the dose volume histograms (DVHs).

A potential way to minimise the impact of the anatomical changes on delivered dose is to utilise adaptive radiotherapy (ART). ART was first proposed by Yan et al. as a treatment method in which changes that occur during radiotherapy can be adapted for in subsequent treatment deliveries [6]. ART has been applied clinically in the brachytherapy part of treatment for cervical cancer [7–9]. The use of ART in the external beam radiotherapy (EBRT) part of treatment is less common and primarily in the form of ad hoc or scheduled replanning for offline ART, or plan of the day libraries for online ART [9]. There have been some investigations and clinical implementation studies into the use of ART for EBRT of cervical cancer [9–13]. However, the majority of these studies have been primarily focussed on adapting to daily variations in the form of a treatment plan library [11–13], without considering long term changes, such as tumour regression. Some methods that have been identified to account for long term changes includes using specific replan triggers or weekly replanning [14–16].

A retrospective study by Lim et al. is a previous study that assessed cervical cancer treatment adaptation based on dosimetric triggers, in which a treatment replan was triggered if one of the dosimetric goals was not met [14]. Other retrospective studies instead performed weekly replanning at pre-determined time points to account for the expected anatomical changes that occur during treatment [15, 16]. Comparisons between weekly replanning or dosimetrically triggered adaptation, compared with no adaptation, have shown improvements in the target coverage and a reduction in dose to the OARs demonstrated with DVH metrics [14–16]. Identification of patients suitable for plan adaptation through imaging would be useful in both offline and online adaptive scenarios, with anatomical changes observed through imaging to be used as potential replan triggers.

This study aimed to identify anatomical changes determined from MRIs that could be used as potential triggers for plan adaptation of EBRT for cervical cancer to achieve dosimetric goals. This was undertaken looking at the relationship between anatomical changes and the resulting dose changes.

## Methods

### Data utilised

The data sourced for this study was retrospective imaging data from 11 patients treated for cervical cancer between 2019 and 2021 at Liverpool and Macarthur Cancer Therapy Centres, Australia. Ethics approval for this study was obtained from the South Western Sydney Local Health District (SWSLHD) Human Research Ethics Committee (HREC) (2019/ETH04391).

The characteristics of this cohort can be seen in Table 1.

**Table 1:** Characteristics of the patient cohort

Characteristic	Value
Age mean (range) (years)	57 (41–79)
*Histologic Type (Number of Patients)*	
Cervical Adenocarcinoma Cervical Squamous Cell Carcinoma Cervical Small Cell Carcinoma	3 7 1
*FIGO Stage: (Number of Patients)*	
IB1 IB3 IIB IIIC1 IIIC2	1 1 4 4 1
*Volumetric Arc Therapy Dose: (Number of Patients)*	
55 Gy/25Fx 45 Gy/25Fx 41.4 Gy/18Fx	6 4 1
Time between treatment start and mid-treatment MRI (range) (days)	23 (12–37)

### Imaging

Images used consisted of a planning CT acquired before treatment, two pre-treatment MRIs, and one mid-treatment MRI (mean 23 days, range 12–37 days). The two pre-treatment MRIs were imaged with different bladder filling statuses, full and empty, and used for the internal target volume (ITV) delineation. The mid-treatment MRI is typically utilised for the planning of high dose rate brachytherapy and preliminary assessment of treatment response. These mid-treatment MRIs had a bladder filling status of “comfortably” full.

The MRIs were acquired using a 3 T Skyra Siemens magnetic resonance imaging scanner (Siemens Medical Systems, Erlangen, Germany) with a 2-dimension turbo spin-echo sequence to generate T2-weighted MRIs. The pre-treatment MRI parameters were: echo time 98 ms, repetition time approximately 10610–10620 ms, slice thickness 3 mm, imaging frequency 123.23 MHz, bandwidth 400 Hz/pixel, flip angle 160°, pixel spacing 0.688 × 0.688mm^2^ and field of view (FOV) of 220 × 220mm^2^. The mid-treatment MRI parameters were: echo time 106 ms, repetition time approximately 9930–11590 ms, slice thickness 2 mm, image frequency 123.23 MHz, bandwidth 405 Hz/pixel, flip angle 120°, pixel spacing 0.703 × 0.703mm^2^ and FOV of 180 × 180mm^2^. The CT images were acquired with a Philips Brilliance Big Bore CT scanner (Philips Medical Systems, Netherlands) with spiral acquisition (slice thickness 2 mm, pixel spacing 1.172 × 1.172 mm^2^ and FOV 600 × 600 mm^2^.

All MRIs were rigidly registered to the planning CT in MIM (MIM Software Inc., v6.9.5). The rigid registration was performed primarily by matching the bony anatomy of the pelvic bones. All registrations were checked by a qualified medical physicist.

### Volume delineation

Two sets of treatment planning volumes were generated; the first based on the information primarily from the pre-treatment MRI, as well as the co-registered pre-treatment CT where necessary. The second set of contours was predominately based on the co-registered mid-treatment MRI, with support from the pre-treatment CT for any structures of interest extending beyond the mid-treatment MRI FOV. These will be referred to as the pre-treatment anatomy/contours and the mid-treatment anatomy/contours, respectively. The original contours of the pre-treatment anatomy were delineated during clinical practice by one of two experienced radiation oncologists (ROs). The mid-treatment volumes were delineated based on the mid-treatment MRI by an RO registrar and were reviewed and edited as necessary by the RO who delineated the original pre-treatment contours for that patient.

The OARs delineated included the bladder, bowel and rectum. The TVs delineated included the gross tumour volume of the tumour (GTV-T), the high-risk and low-risk clinical target volume of the tumour (CTV-T-HR and CTV-T-LR), the low-risk internal target volume of the tumour (ITV-T-LR), and the internal target volume and planning target volume intended to receive 45 Gy (ITV45 and PTV45). The delineation of these volumes was primarily based on information from the MRI, except for the bowel which used both MRI and CT information due to not being completely imaged by the MRI. The definitions for these volumes can be found in the Online Resource 1.

For the pre-treatment contours, the internal target volume (ITV-T-LR) was based on the full and empty bladder MRIs as recommended by the eviQ guidelines [17]. For the mid-treatment MRI however, due to only having one bladder filling status, a margin approach was used by the ROs to delineate the ITV-T-LR instead. A margin based approach is one recommended approach for creation of a PTV from a CTV, with eviQ recommending a non-isotropic expansion of the CTV of 1–2 cm in this case [17, 18]. A similar method was used to create our target volumes. A non-isotropic expansion of 0.5–1 cm from the CTV-T-LR was used to create the ITV-T-LR for the mid-treatment images in this study (see Online Resource 1). The ITV45 was created by combining the ITV-T-LR and any nodal volume delineated from the pre-treatment MRI. The PTV45 was generated with 0.5 cm isotropic expansion from the ITV45. This gave a total non-isotropic expansion from the CTV-T-LR to the PTV45 of 1–1.5 cm, in line with the eviQ recommendations of 1–2 cm.

### VMAT treatment planning

Volumetric modulated arc therapy (VMAT) plans were generated in Pinnacle v16.2.1 (Philips Medical Systems, Fitchburg, Wisconsin, USA) based on the pre-treatment contours. For patients who received a single dose level with no boost for their treatment, a new 45 Gy/ 25 fractions single dose level plan was generated retrospectively (to ensure planning consistency for the study). For patients who received a boost in their original treatment, a new 45 Gy base plan with either 55–57.5 Gy simultaneous integrated boost for 25 fractions was created retrospectively. All plans generated were reviewed and approved by an experienced radiation therapist. DVH metrics were calculated for both pre- and mid-treatment contours.

### Anatomical and dosimetric changes

Anatomical changes to the volumes delineated were assessed using changes in size (cc and %) and change in centroid position in the superior-inferior, posterior-anterior, and lateral (Z, Y, X respectively) directions (Table 2). Some of these metrics were determined in MIM (MIM Software Inc., v6.9.5), whilst others were calculated using basic python code.

**Table 2:** Metrics used to assess anatomical changes with formulas

Metric	Formula
Volume Change (cc)	$\:{\Delta\:}V\:\left(cc\right)=\:{V}_{2}-{V}_{1}$
Volume Change (%)	$\:{\Delta\:}V\left(\%\right)=\frac{{V}_{2}-{V}_{1}}{{V}_{1}}$
Centroid Position Change (X, Y, Z)	$\:{\Delta\:}X={X}_{2}-{X}_{1}$ $\:{\Delta\:}\text{Y}={Y}_{2}-{Y}_{1}$ $\:{\Delta\:}\text{Z}={Z}_{2}-{Z}_{1}$

1– pre-treatment, 2– mid-treatment, V– volume, X,Y, Z = position in lateral, posterior-anterior and superior-inferior direction

The dosimetric changes to the OARs were assessed using the volume receiving 30 Gy and 40 Gy (V30Gy and V40Gy respectively). For the TVs, the change in target coverage was assessed using change in volume receiving 95% and 100% of 45 Gy (V95% and V100% respectively), as well as the dose that 95% and 98% of the target volume received (D95% and D98% respectively). The DVH data was exported from Pinnacle and analysed using PyDicer, an open source, python library (P Chlap et al., (2023) PyDicer, URL: https://github.com/AustralianCancerDataNetwork/pydicer.).

### Analysis– statistical and clinical impact

The mean, standard deviation, maximum value, and minimum value were determined for all anatomical and dose metrics for all patients. Statistical significance was determined using the SciPy python library with the Statistical Functions module (scipy.stats) [19]. The normality of the data was found using the Shapiro-Wilk Test, where a *p*-value > 0.05 was used to determine normal distribution. For data determined to be normally distributed, a paired t-test was applied, whereas, for data not normally distributed, a Wilcoxon-Signed Ranks Test was applied. Statistical significance was determined with a *p*-value < 0.05.

The clinical impact of the changes was assessed based on aims from the local treatment planning protocol (primarily based on EMBRACE II protocol [8]), listed in Table 3. Pre- and mid-treatment DVH values were assessed as per protocol, a minor variation, or a breach.

**Table 3:** Treatment plan aims for the different volumes

Volume	Objective	Per protocol	Minor Variation	Breach
Bladder	V30Gy	< 60%	< 75%	> 75%
	V40Gy	< 75%	< 85%	> 85%
Bowel	V30Gy	< 100 cc	< 250 cc	> 250 cc
	V40Gy	< 350 cc	< 500 cc	> 500 cc
Rectum	V30Gy	< 85%	< 90	> 90
	V40Gy	< 95	< 100	> 100
ITV45	D98%	100	> 95	< 95
	V95%	100	> 98	< 98
	V100%	> 97	N/A	< 97
PTV45	V95%	100	> 95	< 95
	D95%	> 98	> 95	< 95
	V100%	> 90	N/A	< 90

### Potential trigger identification

Anatomical triggers for plan adaptation were identified as anatomical changes that were highly correlated with dosimetric changes. Correlations between anatomical and dosimetric changes were calculated using IMB SPSS Statistics v28.0. (IBM Corp., Armonk, New York, USA). The changes were determined to be highly correlated when the Pearson’s correlation coefficient (R) was 0.9<|R|≤1 and there was a *p*-value < 0.01.

## Results

All numerical values for the anatomical changes that occurred during the treatment of cervical cancer with EBRT, as well as the dosimetric changes determined by this retrospective analysis can be found in the Online Resource 2.

### Anatomical changes

**Fig. 1:**
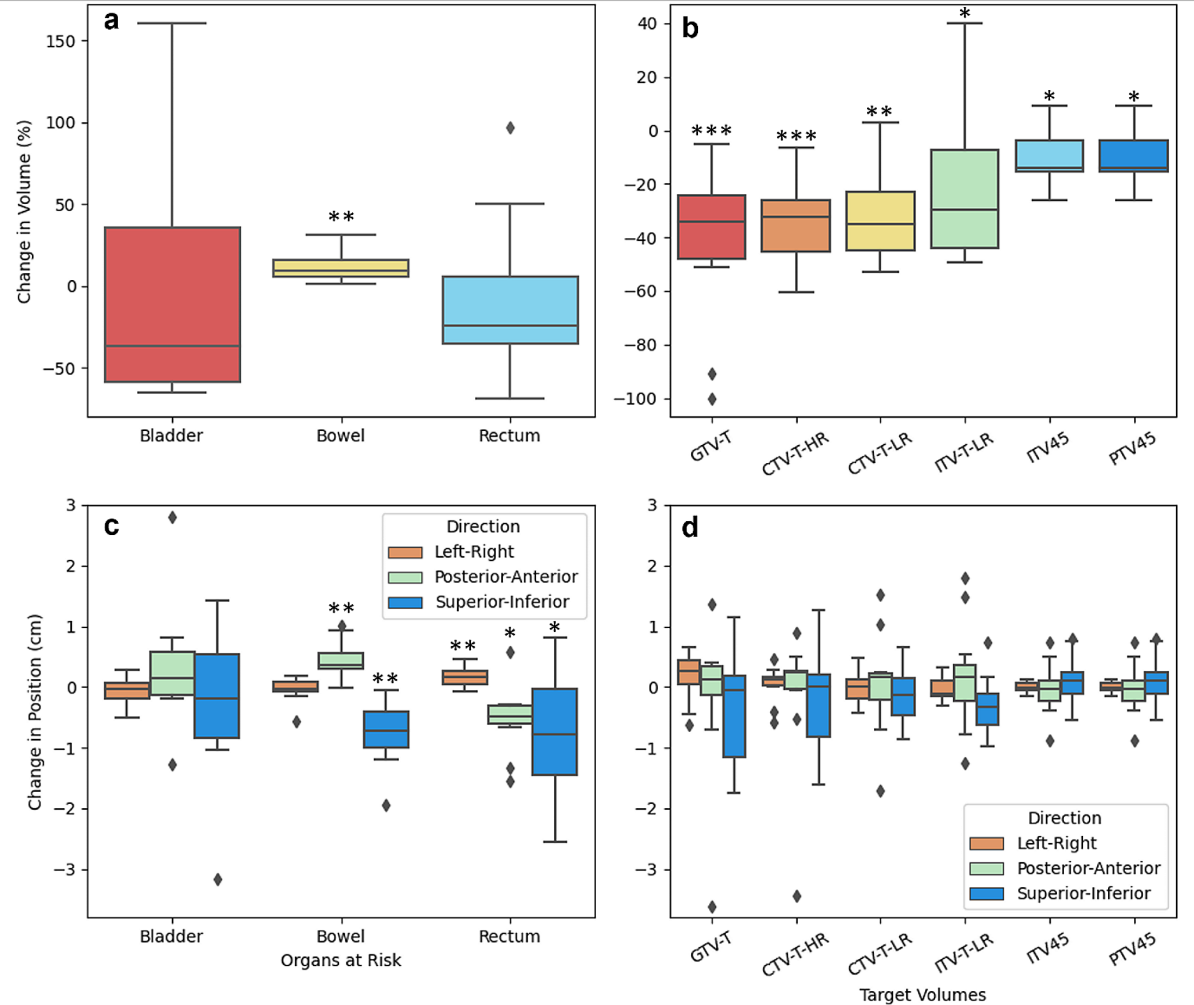
The change in the volume of the OARs and TVs during treatment, a and b respectively, and the changes in position of the OARs and TVs during treatment, c and d respectively. (* = *p* < 0.05, ** = *p* < 0.01 and *** = *p* < 0.001)

The anatomical positional and volume changes are displayed in Fig. 1. The only OAR to experience a statistically significant volume change was the bowel which had an increase in volume of 11.4 ± 8.7% (*p* = 0.001). The largest positional change for the bowel was in the inferior direction, with a mean shift of 0.74 ± 0.53 cm, followed by a shift in the posterior direction of 0.44 ± 0.31 cm, both of which were statistically significant (*p* = 0.001 for both). The rectum saw statistically significant shifts in all directions with shifts inferiorly of 0.75 ± 0.97 cm, anteriorly of 0.52 ± 0.53 cm and laterally of 0.17 ± 0.16 cm, and with *p*-values of 0.035, 0.011 and 0.009, respectively.

All decreases in target volume size were statistically significant. The largest change in volume was observed for the GTV-T, with a mean decrease of 41.5 ± 28.4%, followed by the CTV-T-HR, CTV-T-LR, ITV-T-LR, ITV45 and PTV45. Additionally, for one of the GTV-T cases, the tumour appeared to have had a complete radiological response based on the imaging, therefore the mid-treatment volume was 0 cc, so this volume could not be included for the calculation of other metrics including the positional shift or changes to the DVH metrics.

No positional changes in the TVs were determined to be statistically significant, however, a wide range of positional shifts were observed. For example, as seen in Fig. 1d, the GTV-T had a range of changes between 1.36 cm posteriorly to 3.61 cm anteriorly, as well as changes between 1.14 cm superiorly to 1.74 cm inferiorly. Additionally, from Fig. 1d, positional shifts of above 1 cm in the posterior-anterior direction and superior-inferior direction can be observed for several volumes. The range of positional changes in the lateral direction appears to be less than those of the other directions for all TVs.

**Fig. 2:**
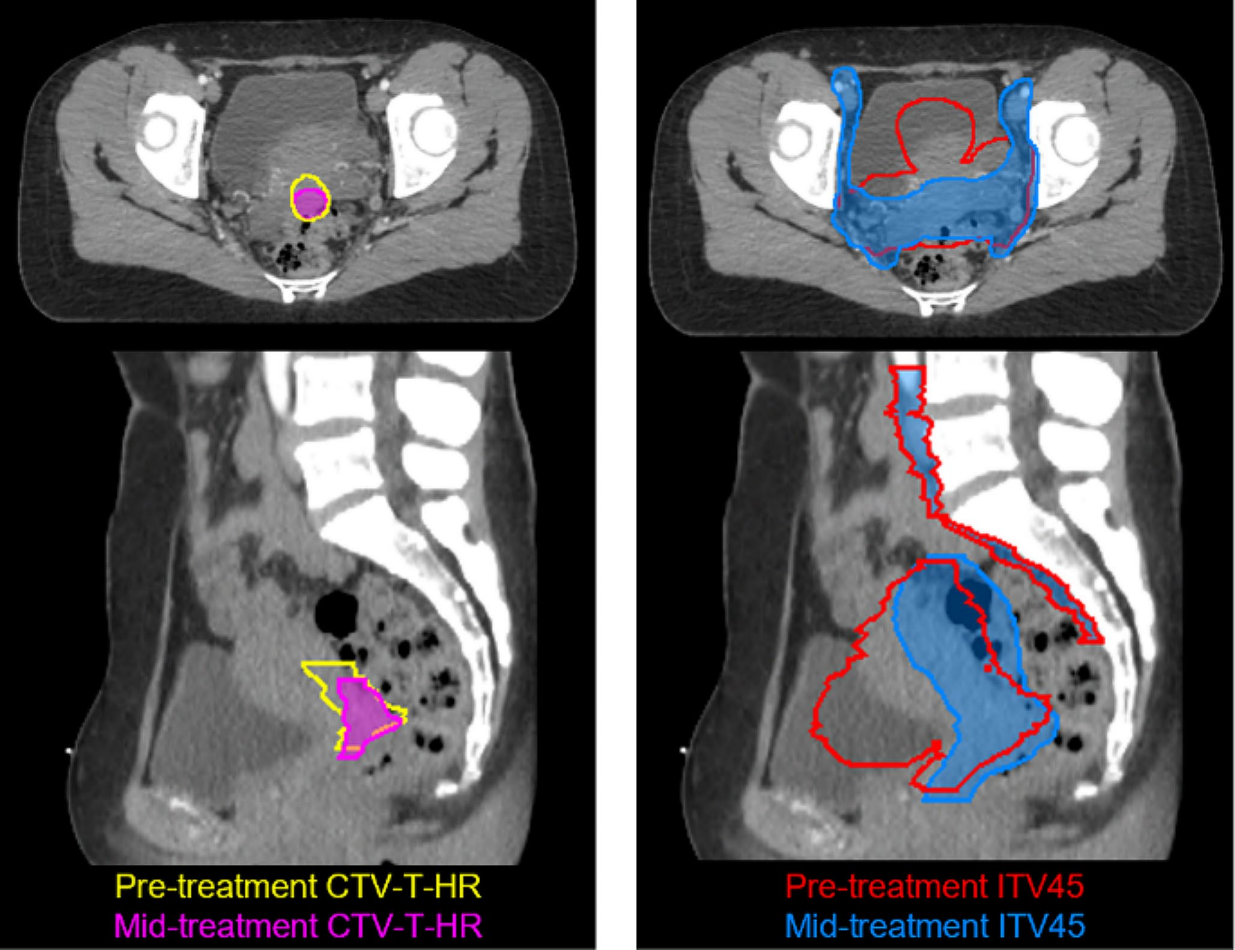
Example CT showing the changes between pre-treatment and mid-treatment contours (CTV-T-HR and ITV45)

An example of some of the anatomical changes that are occurring can be seen in Fig. 2. It can be observed that there is a decrease in size of the CTV-T-HR and the ITV45. However, for the shape of the ITV45, it is very different between the two time points due to the described variation in delineation method, whereas the CTV-T-HR retains a similar shape.

### Dosimetric changes

**Fig. 3:**
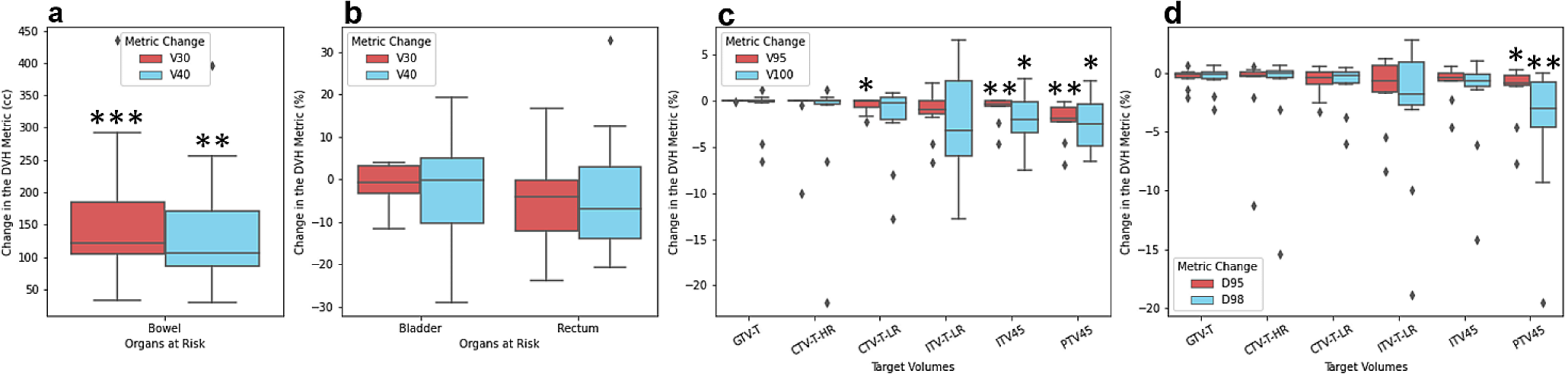
(**a**) Dosimetric changes to the bowel in terms of V30Gy (cc) and V40Gy (cc). (**b**) Dosimetric changes to the OARs in terms of V30Gy (%) and V40Gy (%). (**C**) Dosimetric changes to the TVs in terms of V95% (%) and V100% (%). (**D**) Dosimetric change to the TVs in terms of D95% (%) and D98% (%) (* = *p* < 0.05, ** = *p* < 0.01, *** = *p* < 0.001)

Figure 3 shows the changes to the DVH metrics of the OARs and TVs. Individual volumes experienced varying DVH metric changes. The bowel was the only OAR to have a statistically significant change to its DVH metrics with an increase in V30Gy of 162 ± 110 cc and for V40Gy 145 ± 98 cc, as seen in Fig. 3a. The rectum had the widest range of changes to its V30Gy, whereas the bladder had the widest range of changes of V40Gy, as seen in Fig. 3b. All bowel volumes experienced an increase in dose to the volume, whereas the bladder and rectum both experienced increases and decreases to their doses, with no overall significance.

All TVs experienced a mean decrease in the target coverage DVH metrics of V95%, V100%, D95% and D98% which can be observed in Fig. 3c and d. The smallest changes occurred for the GTV which saw decreases in mean values of less than 1%. The ITV45 and PTV45 had the largest changes in target coverage metrics with mean values between 0.76% and 4.29%. The ITV45 and PTV45 were the only TVs to experience statistically significant decreases in the target coverage metrics of both the V95% and V100%, where the PTV45 also had a statistically significant change in the D95% and D98% (*p* = 0.013, 0.006 respectively). The CTV-T-LR also saw a statistically significant change in the V95% of -0.49 ± 0.77% (*p* = 0.046).

As can be seen in Fig. 3c and d, each of the target volumes had outliers for the different DVH metrics. These represent the volumes that experienced larger changes compared with the mean values. For example, there are two data points in the V100% graph (Fig. 3c) for the GTV-T that have a decrease in target coverage of 4.7% and 6.6%. Additionally, there was also one patient who had a large decrease in the target coverage of the CTV-T-HR as seen by the changes to the V95% and V100% of -10.0% and − 21.9%, respectively.

### Clinical significance

Presented in Table 4 for the OARs and Table 5 for the TVs, is the clinical impact determined by comparing DVH metrics of the OARs, ITV45 and PTV45, with the aims from the treatment planning protocol. For the bladder and rectum, no major clinical changes were observed. This can be seen by a similar number of patients whose DVH metrics were per protocol or in need of minor variation, and no changes to the number of patients in breach of their bladder and rectum aims. The bowel had an increase in number of patients who recorded a breach in their bowel aims from 3 to 6 for the V30Gy (cc) and 2 to 9 for the V40Gy (cc) between the pre-treatment and mid-treatment timepoints.

**Table 4:** Clinical impact of the anatomical changes of the OARs on the DVH aims as assessed by the number of patients in each category (total = 11)

OARs	DVH Metric	Timepoint	Per Protocol	Minor Variation	Breach
Bladder	V30Gy	Initial	2	3	6
		Mid	3	2	6
	V40Gy	Initial	4	4	3
		Mid	4	4	3
Bowel	V30Gy	Initial	5	3	3
		Mid	1	4	6
	V40Gy	Initial	2	7	2
		Mid	0	2	9
Rectum	V30Gy	Initial	9	2	0
		Mid	9	2	0
	V40Gy	Initial	10	1	0
		Mid	9	2	0

Even though treatment plans are designed based on the pre-treatment anatomy, some treatment aims were breached for our initial timepoint. The reason for potential breaches or need for minor variation of some of the TVs is due to the method used to assess the aims for TV as the TV aims are designed for a TV which excludes any overlap with the OARs.

The number of patients in breach of the treatment planning protocol increased between the initial and mid-treatment timepoint for all metrics except V100% which remained the same.

**Table 5:** Clinical impact of the anatomical changes of the TVs on the DVH aims as assessed by the number of patients in each category (total = 11)

TVs	Metric	Timepoint	Per Protocol	Minor Variation	Breach
ITV45	D98%	Initial	3	8	0
		Mid	2	6	3
	V95%	Initial	0	11	0
		Mid	0	8	3
	V100%	Initial	4	N/A	7
		Mid	4	N/A	7
PTV45	D95%	Initial	11	0	0
		Mid	7	2	2
	V95%	Initial	0	11	0
		Mid	0	9	2
	V100%	Initial	4	N/A	7
		Mid	2	N/A	9

### Correlations

The highly correlated relationships between the anatomical changes and dosimetry can be observed in Table 6. The position of the CTV-T-HR in the posterior-anterior direction was found to be highly correlated with all the dosimetric changes in terms of the V95%, D95%, D98% and V100%. The volume of the bowel was highly correlated with the V30Gy (cc) and V40Gy (cc). The position of the bowel in the superior-inferior direction had a negative correlation with the V30Gy (cc) and V40Gy (cc). Although the change in bowel position in the posterior direction was statistically significant, it was not highly correlated with any dosimetric changes. The position of the GTV-T in the posterior-anterior direction was highly correlated with the change in GTV-T V95%.

**Table 6:** Highly correlated anatomical and dosimetric changes

Anatomical Change	Dosimetric Change	Pearson’s Correlation Coefficient, *R*	*P*-value
Bowel Volume (cc)	Bowel V30Gy (cc)	0.981	< 0.001
	Bowel V40Gy (cc)	0.946	< 0.001
Bowel Position, Superior-Inferior Direction (cm)	Bowel V40Gy (cc)	-0.923	< 0.001
	Bowel V30Gy (cc)	-0.921	< 0.001
CTV-T-HR Position, Posterior-Anterior Direction (cm)	CTV-T-HR V95%	0.950	< 0.001
	CTV-T-HR D95%	0.949	< 0.001
	CTV-T-HR D98%	0.947	< 0.001
	CTV-T-HR V100%	0.931	< 0.001
GTV-T Position, Posterior-Anterior Direction (cm)	GTV-T V95%	0.916	< 0.001

### Potential anatomical triggers

Based on the correlation analysis, potential anatomical triggers for plan adaptation to reduce dose delivered to the bowel include:

- Increase in volume of the bowel (cc).
- Change in position of the bowel in inferior direction (cm).

To maintain the target coverage of the CTV-T-HR, the following anatomical trigger could potentially be used:

- Change in position of the CTV-T-HR in the anterior direction (cm).

To adapt a treatment plan to reduce the statistically and clinically significant increases in dose to the bowel, the anatomical triggers of an increase bowel volume or change in bowel position in the inferior direction could potentially be used.

To assess the target coverage of the CTV-T-HR, the position of this volume in the anterior direction should be monitored and potentially used as a trigger to ensure adequate target coverage.

## Discussion

This study investigated the anatomical and retrospective dosimetric changes to the TVs and OARs during the treatment of cervical cancer with external beam radiotherapy. These changes were quantified through delineation of the TVs and OARs prior to treatment commencement and approximately 23 days into treatment. A treatment plan was then created based on the pre-treatment contours and recalculated on the mid-treatment contours to assess dosimetric changes retrospectively. Through correlation analysis between the anatomical and dosimetric changes, potential contour related triggers for plan adaptation were identified. Previous studies have not identified specific anatomical triggers through correlation analysis, but rather have investigated plan adaptation in the form of predetermined replanning timepoints or specific dosimetric triggers [14–16].

Our study showed that for all patients, an increase in bowel volume was observed, as well as positional shifts in the inferior and posterior direction, all of which were statistically significant. The change in volume and the positional shift in the inferior direction were highly correlated with the dosimetric changes to the V30Gy (cc) and V40Gy (cc). These dosimetric changes were both statistically and clinically significant, and likely occurred as the bowel extended inferiorly towards the treatment field during treatment. This suggests that to reduce the increase in dose delivered to the bowel, the volume of the bowel and the position of the bowel in the superior-inferior direction could be used as potential triggers for plan adaptation.

A statistically and clinically significant decrease in the target coverage of the ITV45 and PTV45 was observed in terms of the V95% and V100%. It is important to note that the ITV45 and PTV45 are planning volumes used to ensure the tumour is adequately covered throughout the treatment. No statistically significant changes were observed in the coverage of the GTV and CTVs, indicating the ITV45 and PTV45 mostly succeeded in maintaining clinical target coverage throughout the treatment in most cases. There were, however, 2 cases from 11 where the GTV-T and CTV-T-HR experienced larger decreases in their target coverage.

The position of the GTV-T and CTV-T-HR in the posterior-anterior direction was observed to be highly correlated with the target coverage in terms of V95% for the GTV-T and all target coverage measures for the CTV-T-HR. The shift of these volumes in the anterior direction being highly correlated with a decrease in target coverage is likely due to a positional shift of the volumes towards the bladder, which receives minimal dose compared to the clinical TVs. Therefore, the position of these volumes in this direction could be a potential anatomical trigger for plan adaptation to ensure adequate target coverage throughout treatment.

The dosimetric changes observed in this study, including a decrease in target coverage for the PTV and an increase in dose to the bowel, have also been observed in the literature [5, 14, 16], although in these studies the changes have not been correlated with anatomical changes. Han et al. performed a study using weekly CT images to assess interfractional dose variations [5]. They also observed a decrease in the PTV target coverage and an increase in dose to the bowel at approximately week 3 and week 4 of treatment [5], similar to the timing of the mid-treatment time point in this study.

Lim et al. investigated the difference between plan adaptation based on anatomical changes and dosimetric changes [14]. The anatomical based replan consisted of performing a replan approximately halfway through treatment to account for anatomical changes such as tumour regression, and the dosimetric based replan was performed when target coverage goals were not achieved for a weekly MRI [14]. The anatomical based replan is similar to this study, as although a replan was not generated in our study, we performed a comparison between the pre- and mid-treatment contours [14]. Similarly to the Lim et al. investigation, our study observed an increase in the dose to the bowel and a slight decrease in target coverage, whilst in the Lim et al. study, the anatomical based replan reduced dose to the bowel and slightly reduced target coverage failure [14]. However, in their study, the dosimetric based replan compared to no replanning or the anatomical based replan, proved more effective at maintaining target coverage but showed no benefits for sparing of dose to the OARs [14].

Currently, ART has been implemented clinically for the brachytherapy part of cervical cancer treatment, whereas for the EBRT, studies investigating the implementation of ART for cervical cancer have been primarily focussed on online ART, adapting for daily anatomical variations [9, 10]. Current studies that have investigated offline ART for cervical cancer have been performed retrospectively, with either a single or weekly replan occurring [14–16]. Besides Lim et al.’s dosimetrically triggered replanning method, no specific triggers have been utilised and overall, no anatomical triggers have been identified or used. An anatomical trigger could be determined using only acquired imaging, without the need for generation of a new treatment plan for every image. Our study shows that the changes to the superior-inferior position and volume of the bowel, as well as posterior-anterior position of the CTV-T-HR could be potentially used to trigger treatment replans to improve dosimetric outcomes.

A limitation of this study was the use of retrospective data consisting of a mid-treatment MRI with a smaller FOV that did not image the entirety of the bowel. This resulted in only changes within the FOV visualised. Therefore, the inferior shift of the bowel, which occurred likely due to the decrease in GTV during treatment, resulted in the overall bowel volume increasing. The reason for this increase in bowel volume does not hinder the results obtained as seen by the dosimetric changes measured in this study. Whilst most OARs measure the amount of volume receiving a specific dose as percentage of the total volume (e.g. V30Gy (%)), we measure the bowel volume receiving a specific dose in cubic centimetres (e.g. V30Gy (cc)). Therefore, although the volume of the bowel increases due to imaging and delineation methods, the part of the bowel within the MRI FOV where the volume changes occur, is also the part of the bowel within the treatment field. So, any changes within the MRI FOV are also most commonly the changes within treatment field, therefore our results of dosimetric changes to the bowel are still valid.

Another limitation of this study was the delineation method of the ITV. Since the mid-treatment imaging consisted of only one MRI with an “comfortably” full bladder, the mid-treatment ITV was generated using a margin-based approach. Whereas the pre-treatment ITV was based on the full- and empty-bladder MRIs. This is likely one of the reasons for the decrease in target coverage of the ITV and PTV as the shape of the mid-treatment contour often changed relative to the pre-treatment contours, as seen in Fig. 2. Regardless of the delineation method of the ITV and PTV, the target coverage of the GTV and CTV remained adequate.

Additionally, the sample size of this study was relatively small with data from 11 patients sourced. With an increase in sample size, a better overall picture could be obtained, and correlations between anatomical and dosimetric changes could be further highlighted and strengthened. Furthermore, carrying out this investigation across multiple institutions would further enhance the outcomes observed in this study, although the results in this paper have allowed for valuable insights into some potential triggers of adaptive radiotherapy.

## Conclusion

Changes in bowel dose were seen at treatment mid-point, but not for other OARs. The change in the volume and position of the bowel in the superior-inferior direction and change in position of the CTV-T-HR in the posterior-anterior direction demonstrated high correlation with associated dosimetric changes and could potentially be used as anatomical triggers to determine the need for plan adaptation for cervical cancer patients. This could provide an approach for determining patients who would benefit from a replan.
